# A New Route to Enhance the Packing Density of Buckypaper for Superior Piezoresistive Sensor Characteristics

**DOI:** 10.3390/s20102904

**Published:** 2020-05-20

**Authors:** Mustafa Danish, Sida Luo

**Affiliations:** School of Mechanical Engineering and Automation, Beihang University, Beijing 100191, China; mustafadanish_85@buaa.edu.cn

**Keywords:** carbon nanotubes, buckypaper, structural characterization, piezoresistive sensors

## Abstract

Transforming individual carbon nanotubes (CNTs) into bulk form is necessary for the utilization of the extraordinary properties of CNTs in sensor applications. Individual CNTs are randomly arranged when transformed into the bulk structure in the form of buckypaper. The random arrangement has many pores among individual CNTs, which can be treated as gaps or defects contributing to the degradation of CNT properties in the bulk form. A novel technique of filling these gaps is successfully developed in this study and termed as a gap-filling technique (GFT). The GFT is implemented on SWCNT-based buckypaper in which the pores are filled through small-size MWCNTs, resulting in a ~45.9% improvement in packing density. The GFT is validated through the analysis of packing density along with characterization and surface morphological study of buckypaper using Raman spectrum, particle size analysis, scanning electron microscopy, atomic force microscopy and optical microscopy. The sensor characteristics parameters of buckypaper are investigated using a dynamic mechanical analyzer attached with a digital multimeter. The percentage improvement in the electrical conductivity, tensile gauge factor, tensile strength and failure strain of a GFT-implemented buckypaper sensor are calculated as 4.11 ± 0.61, 44.81 ± 1.72, 49.82 ± 8.21 and 113.36 ± 28.74, respectively.

## 1. Introduction

The discovery of carbon nanotubes (CNTs) by Iijima [1] led to extensive research for the utilization of these unique structures with extraordinary properties in the field of electrical storage devices [2,3], digital electronics devices [4,5], structure health monitoring (SHM) sensors [6,7] and concrete reinforcement applications [8,9], etc. Both theoretically and experimentally, it is proved that CNTs inherit excellent physical properties having an electrical conductivity of 3 × 10^6^ S/m [10,11], the thermal conductivity of 2000–3500 W/m·K [12,13], the mechanical strength of 100 GPa [14], the Young’s modulus of 1 TPa [15,16] and the piezoresistive gauge factor (GF) of 2900 [17,18].

For the utilization of CNTs in SHM applications, it is desired to transform them into bulk form to utilize the superior piezoresistive characteristics of individual CNTs. Buckypaper, which is a thin sheet of micron size thickness made from CNTs, is the most common bulk form used in tensile sensing applications. The buckypaper is usually developed through the hydroentanglement process [19] introduced by Zhang, X. et al., and the hydraulic press method [20] proposed by Siddhant, D. et al., which can be used to develop a large size buckypaper. The vacuum filtration method is preferred by many researchers [21,22,23,24] for developing small size buckypaper in the lab environment with the use of simple filtration apparatus. Moreover, the vacuum filtration process provides a way to tune the buckypaper properties with the variation of dispersion processing parameters.

In the form of buckypaper, the CNTs are randomly arranged [25,26] due to Van der Waal’s attractions. Because of the random arrangement of CNTs, the excellent individual CNTs properties deteriorate significantly in the bulk form of buckypaper, limiting their use in sensors applications. Several studies in the past were done to improve the buckypaper properties. For instance, Min, J. Y. et al. tried to improve the buckypaper properties by functionalizing the raw CNTs using polyvinyl alcohol and showed improvement of 189%, 443% and 166% in mechanical strength, Young’s modulus, and failure strain, respectively, while compromising the electrical conductivity of buckypaper [27]. Many researchers established procedures for the alignment of CNTs in the buckypaper through stretching techniques [28,29,30]. The method of stretching works to improve the sensitivity of the buckypaper-based sensors in terms of improved GF. Still, it reduces the mechanical properties in terms of the lower magnitude of failure strain. A few researchers further enhanced the GF of the CNT-based sensors to several hundred by intentionally creating microcracks in the sensor [31,32]. The introduction of microcracks can enhance the sensitivity of the sensor but compromise the electrical conductivity in the elongated state.

These studies focused on improving a targeted physical property of buckypaper while compromising the others. However, the buckypaper properties can be enhanced altogether, including piezoresistive, mechanical and electrical properties, if the random arrangement of CNTs in the buckypaper could be minimized but not eliminated. One of the features in the random arrangement of CNTs are the existing pores among individual CNTs, which can be found easily through the scanning electron microscope (SEM) surface morphology of buckypaper. These pores can be regarded as gaps or defects resulting in the degradation of the physical properties of CNT-based bulk structure. The elimination/minimization of these gaps can lead to improve the packing density of buckypaper, which can result in significant improvement in the sensing characteristics of buckypaper, including improved piezoresistivity to get superior sensitivity of the sensor, improved mechanical properties to get a higher level of strain monitoring regions and superior electrical conductivity to lower the energy consumption of sensor systems.

The porosity or packing density affects the buckypaper properties [33]; therefore, some researchers studied the magnitude of porosity in buckypaper. As per the recent findings, the porosity of as-fabricated buckypaper obtained from the vacuum filtration method generally ranges between 60%–80% of their total volume [34,35,36]. For superior electrical, mechanical, and thermal properties, less porousness, i.e., buckypaper with a higher magnitude of packing density, is desired. Many researchers have demonstrated through experimental evidence that increasing the packing density of buckypaper increases its flexibility [33], surface uniformity [37], mechanical strength [34,38], electrical conductivity [38] and thermal conductivity [39].

The most commonly adopted method for improving the packing density of buckypaper is to apply compressive pressure to buckypaper after its fabrication [33,34]. This method is useful, but the percentage improvement in packing density is limited to a single digit. Some researchers tried to mix metal nanoparticles in CNT buckypaper to improve its packing density [40,41,42]. These methods enhanced the electrical and thermal conductivities of buckypaper but consequently increased the weight of buckypaper. Furthermore, Jianwei Z. et al. showed an improvement of 1.9% in the packing density of buckypaper through pressurized filtration of buckypaper [33]. However, the pressurized filtration technique needs more energy input as well as complicated setup; therefore, it is more expensive than the vacuum filtration technique.

In this study, a novel technique of filling the pores of CNT-based buckypaper, termed as the gap-filling technique (GFT), is presented. The GFT is established through the mixing of two different types of CNTs, differing in their dimensions. Many other researchers have also fabricated buckypapers by mixing different types of CNTs, for instance, Shu li et al. mixed multi-wall carbon nanotubes (MWCNTs) in single-wall carbon nanotubes (SWCNTs) at a weight ratio of 3:1 for utilizing buckypapers in liquid sensing applications [43]. The thickness of the resultant buckypaper increased from ~15 to ~25 µm after the addition of MWCNTs, but no information is provided for the packing density of the buckypaper. There are a variety of CNTs available commercially differing in their dimensions. It is demonstrated through the series of experiments, in this study, that it is not the mixing of CNTs that improves the packing density of resultant buckypaper, but it is the mixing of “properly selected” CNTs that improves the packing density through filling the pores of the buckypaper i.e., not all types of CNTs can be used to fill the pores of the buckypaper. The dimensions and the quantity of gap-filler CNTs are the two essential parameters for the improvement of the packing density of the buckypaper. The novelty of GFT is based on the establishment of a generalized criterion for the selection of the dimensions and quantity of gap-filler CNTs, resulting in the improvement of buckypaper sensing characteristics. The criterion is generalized through a series of experimentation and characterization techniques, so GFT can be implemented to improve the packing density of buckypaper fabricated from any large length CNTs. As per our knowledge, it is a new attempt to establish a generalized technique through microstructural characterization for the improvement of buckypaper packing density and modulation of its piezoresistive properties.

In this study, the CNT-based buckypapers are fabricated using the vacuum filtration process, and the pores of the bulk structure of CNTs are filled with the addition of a small quantity of shorter size CNTs, resulting in the significant improvement of packing density. The reduction in defects through the GFT is demonstrated through the analysis of packing density, Raman spectrum, SEM, and atomic force microscopy (AFM) results of resultant buckypaper. The selection of gap-filler CNTs is made based on its dimensions. In GFT, a straightforward rule for the selection of quantity and dimensions of gap filler is demonstrated through careful observation of variation in thickness of resultant buckypaper. It is concluded through series of experiments that the amount and dimension of gap-filler CNTs should be such that it should not increase the thickness of the original buckypaper. Meaning that the gap-filling CNTs should be trapped in the pores of the buckypaper; or, in other words, the gap-filling CNTs should only consume to increase the packing density of resultant buckypaper. 

As a representative case of GFT, in this study, the pores of SWCNT-based buckypaper are filled through the addition of shorter size MWCNTs. In this representative case, the packing density of resultant buckypaper was increased by ~45.9%, resulting in a tremendous improvement in buckypaper-based sensor-related physical properties with a percentage improvement of 4.11 ± 0.61, 44.81 ± 1.72, 49.82 ± 8.21 and 113.36 ± 28.74 in electrical conductivity, tensile GF, tensile strength and failure strain, respectively. 

We believe that the superior sensing characteristics of buckypaper, obtained through the implementation of GFT, can overcome the current industrial challenges regarding inferior sensing characteristics of CNT-based sensors. The enhanced sensor characteristics can open new avenues of using CNTs buckypaper based sensors commercially in industrial applications. 

## 2. Materials and Methods 

### 2.1. Materials and Equipment

Both SWCNTs and MWCNTs used in this study were procured from Time Nano China Inc, having a supplier-claimed purity of 98%. Surfactant triton X-100 was procured from Sigma Aldrich. Ultrasonic sonicator with 480 W of power was used to prepare the dispersion. Eppendorf 5418 centrifuge was used at 5000 rpm for the removal of massive particles from the dispersion. PVDF (polyvinylidene fluoride) membranes were procured from Millipore and were used to filter the CNTs in a filtration set up. CNTs were characterized by Raman spectroscopy and particle size analysis (PSA). The buckypapers were characterized by a morphological study using SEM, AFM, and optical microscopy. Raman spectroscopy was performed in the Horiba Scientific RM HR Evolution equipment. SEM was performed in the JEOL JSM-7001F microscope. PSA was done in Malvern Zetasizer Nano ZS90 equipment. AFM was performed in the Bruker microscope integrated with Fast Scan Asyst software for analysis. Optical microscopic images for measuring the thickness of buckypaper were taken using the Nikon microscope. Universal Laser System DLS 2.3 was used for cutting the buckypaper to prepare the specimen for tensile testing. A dynamic mechanical analyzer (DMA) (Discovery 850, TA Instruments Inc.) was used to determine the physical properties of the buckypaper specimen. A Keithley DMM7510 digital multimeter with LabVIEW interface was used to measure the resistivity of buckypaper and change in resistance during tensile stretching in DMA.

### 2.2. Fabrication of Buckypaper

The CNT-based buckypapers were fabricated using the vacuum filtration process in which a well-homogenized dispersion of CNTs was filtered in the filtration setup with PVDF membranes at a vacuum pressure of 0.95 bar. The dispersion preparation parameters, which include the ratio of CNTs, surfactants and de-ionized (DI) water in dispersion, the parameters of sonication and centrifugation and drying conditions of buckypaper after filtration, have a significant influence on resultant buckypaper properties. Therefore, a well-homogenized dispersion preparation was necessary to achieve superior properties of CNTs in the bulk structure of buckypaper. 

**Figure 1:**
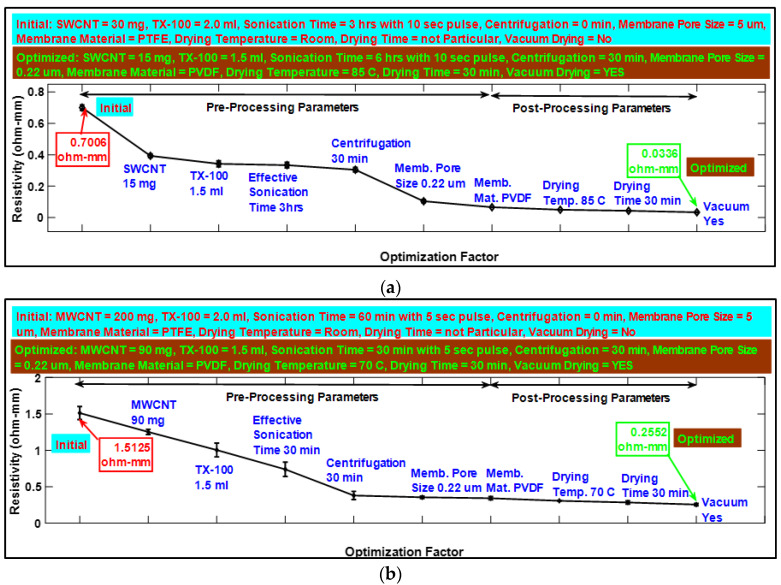
(**a**,**b**) depict, respectively, a summary of the pre- and post-processing optimization parameters for single-wall carbon nanotube (SWCNT)- and multi-wall carbon nanotube (MWCNT)-based buckypapers.

In this study, the CNT dispersion parameters were first optimized to get minimum resistivity (maximum conductivity) of buckypapers, to minimize the energy consumption of sensor systems. Through series of optimization experiments, summarized in Figure 1a,b, it was found that minimum resistivity of ~0.0336 ohm – mm (conductivity = ~29,800 S/m) and ~0.2552 ohm – mm (conductivity = ~4000 S/m) for SWCNT- and MWCNT-based buckypapers, respectively, can be achieved when 75 mL/70 mL of SWCNT/MWCNT, having following dispersion optimization parameters, filtered in vacuum filtration setup with PVDF membrane:

- For SWCNT: 15 mg SWCNT mixed in 1.5 mL Triton X-100 and 100 mL DI Water, sonicated for 6 h with 10 s on/off pulse, centrifuge for 30 min at 5000 rpm and, after filtration, dried in vacuum pressure at 85 °C.
- For MWCNT: 90 mg MWCNT mixed in 1.5 mL Triton X-100 and 100 mL DI Water, sonicated for 1 h with 5 s on/off pulse, centrifuge for 30 min at 5000 rpm and, after filtration, dried in vacuum pressure at 70 °C.

The dispersion optimization experiments were performed by optimizing a single factor at a time. The experimental data of these optimization experiments have been provided in tabular form in supporting materials as Tables S1–S6.

The mixing of CNTs, in this study, was done in the dispersion stage. For the purpose of establishing GFT, the mixing of CNTs was performed as 90% SWCNT and 10% MWCNT, 75% SWCNT and 25%, 50% SWCNT and 50% MWCNT and 25% SWCNT and 75% MWCNT. Here, the ratio % refers to the volumetric % of the dispersion prepared for optimized dispersion parameter of respective CNTs mentioned above, e.g., 90% SWCNT means 90% of 75 mL SWCNT optimized dispersion, i.e., 67.5 mL, similarly, 10% MWCNT means 10% of 70 mL MWCNT optimized dispersion, i.e., 7 mL.

### 2.3. CNTs and Buckypaper Characterization

Raman spectroscopy was adopted to characterize the CNT size and quality in the buckypaper. Raman spectrum was obtained with a 785-nm laser with a 3-mW power source as an excitation light source [44]. Moreover, the contents of SWCNTs in the buckypaper was characterized by the RBM intensity of the Raman spectrum. The particle size analysis (PSA) of the CNTs in the dispersion was performed using a dynamic light scattering technique at a scattering angle of 90 degrees. This technique measures the diffusion of particles moving under Brownian motion and converts this to size and size distribution. The concentration of CNTs in the dispersion was the critical factor for size estimation in PSA. Therefore, the concentration of CNTs in the dispersion was kept at a constant level of 0.15 gm/L. The surface morphology of buckypapers fabricated through the variation of CNTs ratio was studied using SEM. The surface defects in terms of surface roughness and peak-to-peak depth due to gaps or pores among randomly arranged CNTs in buckypaper were analyzed using AFM. The surface defects were estimated at three randomly selected regions of buckypaper, having an area of 10 µm × 10 µm. The buckypaper thickness, which was regarded as a critical indicator for determining the implementation of GFT, was measured with the optical microscope.

### 2.4. Sensor Characteristics Parameters

The impact of GFT on the sensing characteristics of buckypaper was studied in DMA, which was electrically connected with digital multimeter having an interface with LabVIEW. The sensing characteristics of buckypaper considered in this study were, electrical conductivity, GF, tensile strength, and failure strain. The buckypaper was cut into a rectangular shape specimen using Universal Laser System and mounted on the DMA clamps. This rectangular specimen was considered as a strain sensor without any further processing due to the intrinsic piezoresistive and the self-supporting nature of buckypaper. The sensor was then stretched in DMA at a constant strain rate of 0.3%/min from 0.05% strain to failure strain. The sample electrical conductivity was measured before stretching, for which the dimensions of the sample were measured using a Vernier caliper (length and width) and optical microscope (thickness), and the resistance was measured using a digital multimeter. The GF of the sensor was measured with the data of change in resistance during tensile stretching provided by LabView and DMA software. The tensile strength and failure strain were measured from DMA interface software. To avoid human errors, three samples were used, and their readings were averaged.

## 3. Results and Discussion

### 3.1. Characterization of CNTs with Particle Size Analysis

**Figure 2:**
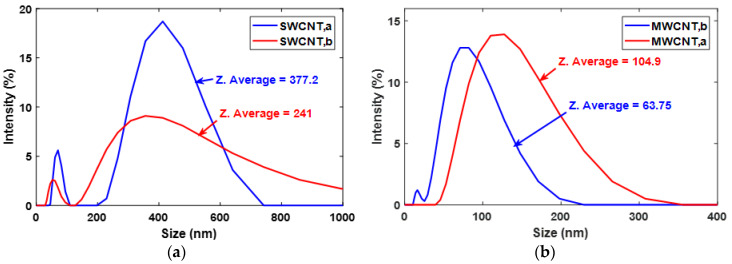
(**a**,**b**) depict the particle size estimation of SWCNTs and MWCNTs, respectively, in a dispersion concentration of 0.15 gm/L.

In this study, two types of SWCNTs and MWCNTs were initially considered. The particle size of SWCNT and MWCNT (both types) was analyzed in the dispersion using a dynamic light scattering technique. The Z-average size, which is also called cumulants mean, is the primary and most stable parameter used in the dynamic light scattering technique, defining the average size of particles in dispersion as a sphere [45]. For the samples having a high aspect ratio like CNTs, a single Z-value may hugely be misleading in terms of considering it as length or diameter of the tube. However, if the bulk magnitude of Z-value readings has been plotted against the intensity of occurring, i.e., a plot of particle size distribution, then the length and the diameter of the CNTs can be estimated. The maximum value obtained from the distribution plot can be considered as the approximate length, and the minimum value can be considered as the approximate diameter of the tube. As the length of individual CNT can vary in the dispersion; moreover, there are possibilities of CNTs agglomerations; therefore, instead of taking the maximum value, we, in this study, have used Z-average value to estimate the length of the CNTs and its agglomerations in the dispersion. As shown in Figure 2a,b, the two types of SWCNTs and MWCNTs differ in their sizes significantly. The Z-average value of SWCNTs and MWCNTs was found to be ~377 and ~241 and ~104 and ~63, respectively. Accordingly, the longer length SWCNT having a Z-average value of ~377 was termed as SWCNT,a, and the shorter length SWCNT having Z- average value of ~241 was termed as SWCNT,b. Similarly, the MWCNT having a Z-average value of ~104 was termed as MWCNT,a, and the MWCNT having a Z-average value of ~63 was termed as MWCNT,b. It was also observed from PSA that the variation in the sizes of SWCNT,b was higher than SWCNT,a, MWCNT,a, and MWCNT,b. The PSA reports have been provided as supporting material.

### 3.2. Characterization of CNTs with Raman Spectrum

**Figure 3:**
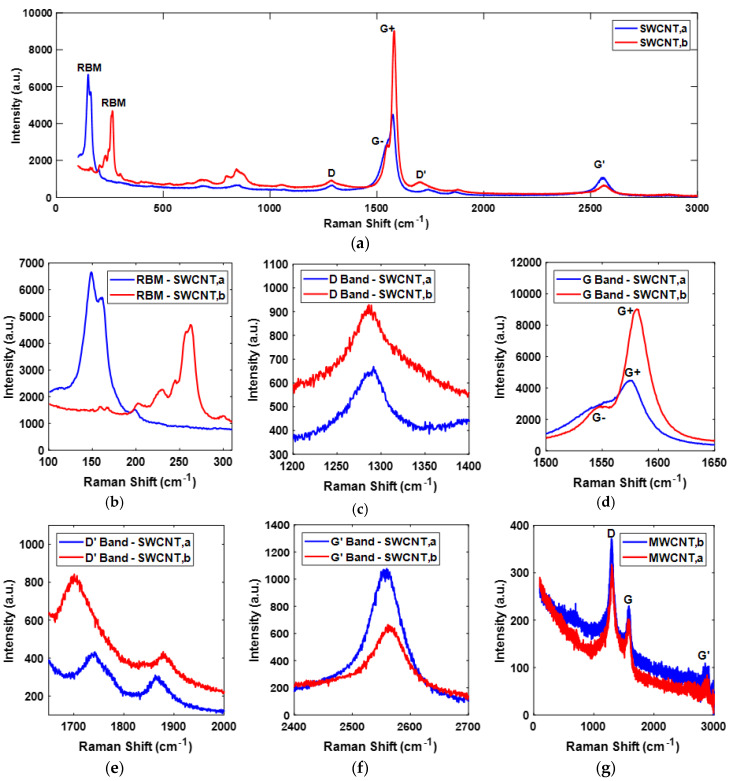
(**a**,**g**) depict the Raman spectrum of SWCNTs and MWCNTs, respectively, between the Raman shift of 100 to 3000 cm^−1^. (**b**–**f**) show the radio breathing mode (RBM), D, G, D’ and G’ bands comparison of SWCNTs, respectively.

As shown in Figure 3a, the Raman spectrum of SWCNT,a and SWCNT,b, matches with the previous studies [44,46,47]. The radio breathing mode (RBM) is important in characterizing the diameters of the tubes [48]. The diameter of SWCNTs are inversely related to RBM frequency as ω_rbm_ (cm^−1^) = (223.5 (cm^−1^)/d_t_ (nm) + 12.5 (cm^−1^) [49]. For graphite structures, there exists a D band that describes the breakdown of symmetry due to the presence of defects in CNTs. The defects can be due to vacancies, edges, or any other discontinuity [48,50]. These defects lower the crystal symmetry of the quasi-infinite lattice of CNTs. The G band in CNTs is due to the shear stretching of carbon bonds in the tube [46]. For SWCNTs, the G band has two peaks, which are generally described by G- and G+ peaks. The G- and G+ peaks correspond to atomic displacements along the circumferential direction and the tube axis, respectively [51]. The G- peak provide a crucial information for characterizing the SWCNTs as metallic or semiconductor type. A broad and dominantly visible G- peak in terms of a substantial downward shift in intensity appears for metallic SWCNTs [52]. The G- band is also related to the diameter of the tubes. Comparatively, the more softened G- band refers to the smaller diameter of the tube [53]. The intensity ratio of the D band and G+ band, i.e., the I_D_/I_G_ ratio, is usually used as the indicator of defects in CNTs; the greater the ratio, the greater the number of defects [54]. The M band, also called D’ band, is related to the defects in the crystalline structure of CNTs specifically related to edge defects. The D band is related to the intervalley mechanism, and D’ band is related to the intravalley mechanism [55]. The G’ band is considered as the overtone of the disorder-induced D band. It is taken as an intrinsic property of the nanotubes and graphite. The G’ band does not require the presence of defects and is also observed in highly crystalline carbon structures, i.e., it is present even when CNTs are defect-free (without D band) [48].

The presence of RBM, in which the atoms of CNT vibrate in the radial direction, was a confirmation of the presence of SWCNTs. The RBM of SWCNT,a appears at a lower frequency and higher excitation than SWCNT,b as shown in Figure 3b, emphasizing that SWCNT,a have larger dimensions as compared to SWCNT,b. From the relationship of SWCNT diameter with RBM frequency, the diameter of SWCNT,a and SWCNT,b was calculated to be ~1.62 and ~0.91 nm, respectively. In both SWCNTs, as shown in Figure 3c,d, there exist D band and G band. The I_D_/I_G_ ratio of SWCNT,a and SWCNT,b was calculated to be ~0.148 and ~0.101, respectively. The calculated I_D_/I_G_ ratios were lower than mentioned in recent studies [46,54], showing that both types of SWCNTs used in this study were of good quality. From the careful observation of G band in Figure 3d, it can be seen that the G- band for both SWCNT,a and SWCNT,b did not have a substantial downward shift of intensity; therefore, both SWCNTs, considered in this study, can be regarded as semiconductor type. Moreover, the G- peak of SWCNT,b was softened as compared to SWCNT,a, confirming that the SWCNT,b was smaller in size than SWCNT,a. These results are in line with the PSA estimations described above. The presence of D’ and G’ bands, as shown in Figure 3e,f, respectively, at the respective Raman shift for CNTs was validating that both SWCNT,a, and SWCNT,b samples were CNTs.

As shown in Figure 3g, the Raman spectrum of two initially considered types of MWCNTs matches with the Raman spectrum of MWCNT mentioned in recent studies [54,56,57]. The absence of RBM mode and presence of D, G, and G’ bands at the respective Raman shift of CNTs confirms the presence of MWCNTs. Due to the superior wall strength of MWCNTs, as compared to SWCNTs, there have minimal likelihood of radial vibrations for carbon atoms in MWCNTs, resulting in the absence of RBM mode [58]. The I_D_/I_G_ ratio of MWCNT,a and MWCNT,b was calculated to be ~1.54 and ~1.63, respectively, better than mentioned in recent work for pristine MWCNT [56], signifying the good quality of both types of MWCNTs, considered in this study.

It is important to note here that the I_D_/I_G_ ratio of SWCNTs is superior than that of MWCNTs. This critical observation will later be used in this study for the validation of GFT.

### 3.3. Surface Morphology of SWCNTs

**Figure 4:**
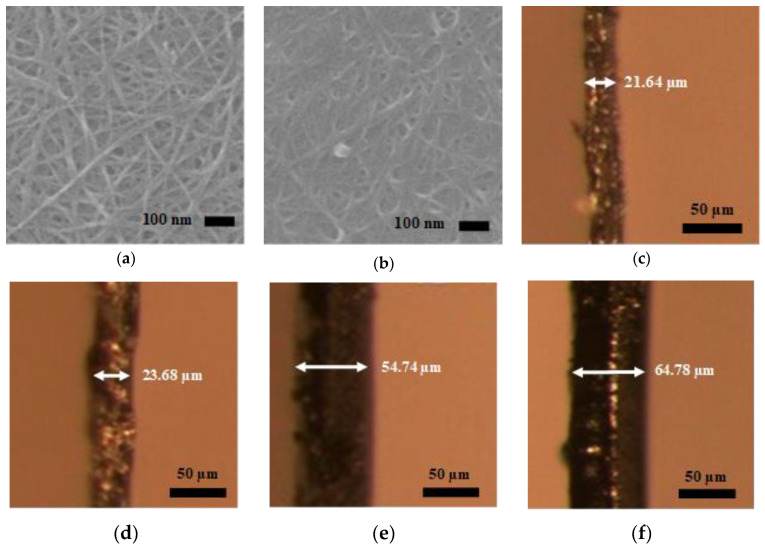
(**a**,**b**) depict the scanning electron microscopy (SEM) of SWCNT,a and SWCNT,b, respectively. The magnitude of pores in SWCNT,a is comparatively higher than SWCNT,b. (**c**–**f**) show the optical microscope images of minimum achievable buckypaper thickness fabricated using SWCNT,a, SWCNT,b, MWCNT,a and MWCNT,b, respectively, having a Z-average value of ~377 nm, ~241 nm, ~105 nm and ~63 nm, respectively.

After the characterization of CNTs with PSA and Raman spectroscopy, the surface morphology was studied using SEM. In the layer-by-layer arrangement of CNTs in buckypaper, only the surface layer of CNTs appears brighter. The dark spots in the SEM image represent vacant spaces or porosity in the surface layer of CNTs; however, there could be CNTs present beneath the dark spots located in the inner layer of CNTs. As shown in Figure 4a,b, both types of SWCNTs were randomly arranged in buckypaper. The gaps or the pores among the random arrangement of SWCNTs were clearly visible. It can be observed from Figure 4a,b that pores in the SWCNT,a structure were higher in magnitude than SWCNT,b, which was possibly due to comparatively large size of SWCNT,a, as analyzed during particle size estimation. This hypothesis was confirmed by the study of Shunsuke et al., where he experimentally showed that the buckypaper fabricated from larger length CNTs has higher porosity and thus a lower packing density as compared to the buckypaper fabricated from smaller length CNTs [57].

### 3.4. GFT Implementation on SWCNT,a

From SEM evidence, supported by PSA, it was concluded that the magnitude of pores in buckypaper was directly related to the length of individual CNTs. This meant that the buckypaper fabricated using longer length CNTs possesses a greater extent of pores than the one fabricated using shorter length CNTs. Based on these experimental observations, one might think of synthesizing buckypapers with shorter length CNTs to get higher packing density thus, superior buckypaper properties. However, it was observed that for the same density, the buckypaper fabricated with larger length CNTs have superior properties as compared to the one fabricated with shorter length CNTs [38]. Moreover, as in the random arrangement of CNT in buckypaper, the individual CNTs were interwoven, making the buckypaper mechanically strong. The longer length CNTs have a greater extent of interlacement, thus providing enough strength to get a free-standing buckypaper with much less amount of CNTs as compared to smaller length CNTs. Therefore, we can get a very thin sheet of buckypaper, having sufficiently fewer mass and superior buckypaper properties, with larger length CNTs as compared to the smaller length CNTs. In other words, we can say that (also observed during dispersion parameters optimization experiments) the minimum achievable buckypaper thickness (MABT) was directly related to the length of the CNTs. As shown in Figure 4c–f, the buckypapers which were fabricated using large length CNTs (in this study SWCNTs) have a smaller MABT as compare to the one fabricated from shorter length CNTs (in this study MWCNTs).

On the one hand, the buckypaper fabricated from larger length CNTs has characteristics like smaller MABT (less mass) and higher mechanical strength and failure strain due to the greater extent of interlacement. On the other hand, it increases the defects in terms of pores among individual CNTs. Therefore, this study focuses on filling up the pores of the buckypaper fabricated using large size CNTs, i.e., SWCNT,a. Although, SWCNT,b have larger Z-average value than MWCNTs, as shown in PSA, but there exist higher degree of variation in the dimensions of SWCNT,b; therefore, SWCNT,b was not used further in this study. The filling of the pores of SWCNT,a will not only improve the packing density, resulting in the improvement of mechanical strength, failure strain, and electrical conductivity, but will also enhance the piezoresistive sensitivity of the sensor because of additional inter-tube interactions.

### 3.5. Selection of Gap-Filler CNTs for SWCNT,a Buckypaper

**Figure 5:**
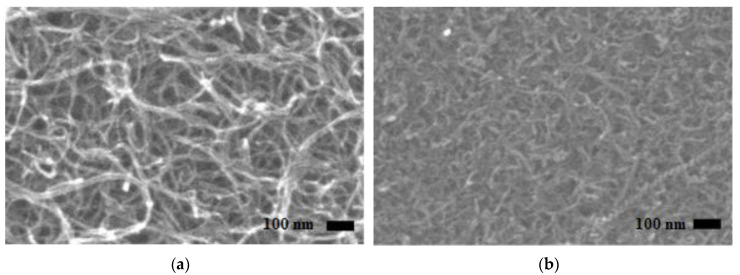
(**a**,**b**) depict the SEM of MWCNT,a and MWCNT,b, respectively. The magnitude of pores in MWCNT,a is comparatively higher than MWCNT,b, suggesting the suitability of MWCNT_,b_ as a gap filler.

The basic idea of this study was to investigate a technique through which the pores of the buckypaper can be filled with smaller size CNTs. For this purpose, after the selection of SWCNT,a-based buckypaper for GFT implementation, it was, now, necessary to investigate the suitable type of CNTs that can be utilized as gap-filler for SWCNT,a-based buckypaper. The surface morphology of smaller size CNTs, i.e., MWCNT,a and MWCNT,b in the form of buckypaper was studied for judging the suitability of these CNTs as gap-filler for SWCNT,a buckypaper. From the SEM observation of MWCNT,a and MWCNT,b buckypapers, as shown in Figure 5a,b, a higher magnitude of pores was observed in MWCNT,a, having a higher Z-average value as compared to MWCNT,b. From this crucial observation, initially, MWCNT,b, having a Z-average value of ~63 nm, was selected as a gap filler for SWCNT,a. For comparison purposes and to validate the results, we also tried to use MWCNT,a as gap-filler.

### 3.6. Investigating the Amount of Gap-Filler MWCNT,b for SWCNT,a Buckypaper

**Figure 6:**
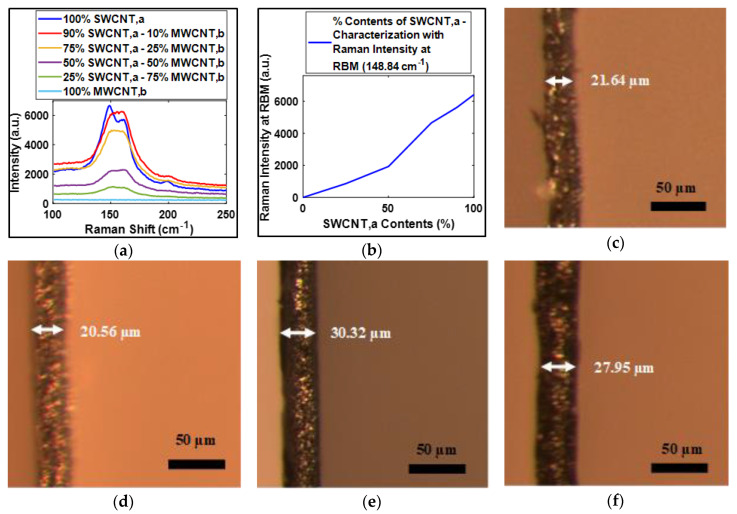
(**a**) RBM mode illustration, showing the depletion of intensity with increasing MWCNT contents. (**b**) Characterization of SWCNT,a contents with Raman intensity at RBM. (**c**–**f**) are optical microscope images of the thickness of buckypapers fabricated with 100% SWCNT,a, 90% SWCNT,a and 10% MWCNT,b, 75% SWCNT,a and 25% MWCNT,b and 90% SWCNT,a and 10% MWCNT,a, respectively.

For the selection of the amount of gap-filler CNTs, a series of experiments have been designed to investigate the amount of MWCNT,b required to fill the pores of SWCNT,a buckypaper. The MWCNT,b in dispersion was mixed with increasing volumetric percentage in SWCNT,a. The buckypapers were fabricated using vacuum filtration process for 90%, 75%, 50% and 25% SWCNT,a with 10%, 25%, 50% and 75% MWCNT,b, respectively. A very interesting feature of Raman spectrum RBM intensity was observed during the experiments of investigating the amount of gap-filler MWCNT,b. Figure 6a shows the variation of RBM with variation in volumetric contents of MWCNT,b. It can be seen that the intensity of RBM mode decreases with the increasing amount of MWCNT,b, which completely vanished for 100% MWCNT,b-based buckypaper. From RBM intensity dying out with increasing MWCNT,b contents, the characterization of the quantity of SWCNT,a in the buckypaper can be done as shown in Figure 6b.

To increase the packing density or for filling up the pores of SWCNT,a buckypaper, the quantity of gap-filler MWCNT,b, should be such that it should not contribute to the thickness of buckypaper. This means that the thickness of buckypaper before and after the addition of gap-filler CNTs should remain at the same magnitude, contribute to increasing the packing density of resultant buckypaper. As shown in the optical microscope images of buckypaper thickness, Figure 6c,d, it was quite evident that when 10% of MWCNT,b was added in SWCNT,a, the thickness of the buckypaper did not increase. When the amount of MWCNT,b was further increased to 25%, the resultant buckypaper thickness significantly increased, as shown in Figure 6e. These results emphasize that 10% of MWCNT,b was sufficient for filling up the pores of SWCNT,a. Any additional amount of MWCNT,b contribute to the thickness of buckypaper after filling up the pores. Moreover, when it was attempted to fill up the pores of SWCNT,a buckypaper, using 10% MWCNT,a, the thickness of buckypaper increased, as shown in Figure 6f. This observation was suggesting that instead of filling the pores, MWCNT,a might have dispersed randomly over the SWCNT,a network due to its comparatively large size. Table S7 in the supporting materials summarizes the experimental data of the buckypaper thickness for the considered buckypaper compositions.

### 3.7. Validation of GFT

An essential aspect of the validation of the current study of GFT was the reduction in pores or gaps through improvement in packing density of SWCNT,a-based buckypaper with the addition of 10% MWCNT,b (gap-filler). The GFT was validated through the analysis of packing density supported with SEM, Raman spectrum, and AFM observations of resultant buckypaper.

**Figure 7:**
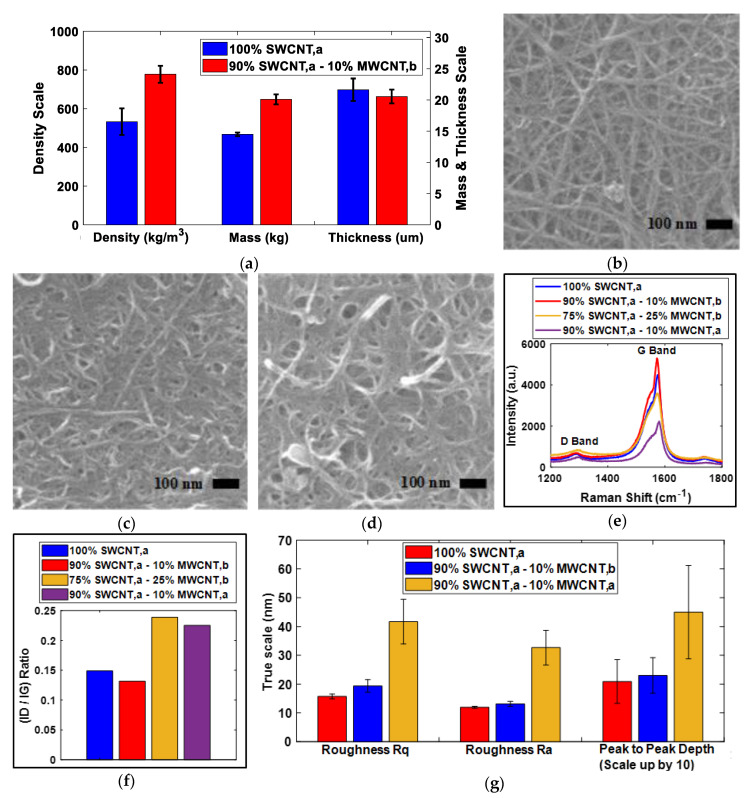
(**a**) Packing density analysis along with mass and thickness of resultant buckypaper fabricated from similar dispersion preparation, filtration process and drying conditions. (**b**–**d**) show SEM images of buckypapers fabricated from 90% SWCNT,a and 10% MWCNT,b, 75% SWCNT,a and 25% MWCNT,b and 90% SWCNT,a and 10% MWCNT,a, respectively. (**e**) Raman spectrum D and G bands of buckypapers fabricated from 100% SWCNT,a, 90% SWCNT,a and 10% MWCNT,b, 75% SWCNT,a and 25% MWCNT,b and 90% SWCNT,a and 10% MWCNT,a, respectively. (**f**) shows the I_D_/I_G_ ratio of respective buckypapers showing a reduction in defects in 90% SWCNT,a and 10% MWCNT,b due the implementation of the gap-filling technique (GFT). (**g**) Atomic force microscopy (AFM) results showing an increase in surface defects when MWCNT,a was used as gap filler.

With the implementation of GFT, as shown in Figure 7a, the packing density of resultant buckypaper, fabricated with 90% SWCNT,a and 10% MWCNT,b, was significantly improved as compared with buckypaper fabricated with 100% SWCNT,a. The mass of the buckypaper was increased from 14.5 to 20.1 mg after GFT implementation. However, the thickness of the buckypaper before and after the GFT implementation remained at approximately the same magnitude, resulting in an improvement of ~45.9% in packing density. This improvement was confirming that pores of buckypaper were filled with the implementation of GFT.

To support and to validate the improvement in packing density after GFT implementation, the morphology of the buckypapers, fabricated from the compositions of 100% SWCNT,a (buckypaper without GFT implementation), 90% SWCNT,a and 10% MWCNT,b (buckypaper with GFT implementation), 75% SWCNT,a and 25% MWCNT,b (buckypaper with an additional amount of gap-filler) and 90% SWCNT,a and 10% MWCNT,a (buckypaper with larger size gap-fillers), were studied using SEM, Raman spectrum and AFM.

As shown in Figure 7b, the surface morphology of buckypaper with GFT implementation was very much similar to the surface morphology of the buckypaper without GFT implementation, as previously shown in Figure 4a. The similarity in morphology was signifying that a small amount of (10%) MWCNT,b was utilized only to fill the pores in between the layers of SWCNT,a buckypaper resulting in the increase of packing density. Furthermore, from the morphological study of buckypaper fabricated from 75% SWCNT,a and 25% MWCNT,b, and 90% SWCNT,a and 10% MWCNT,a, as shown in Figure 7c,d, the surfaces resembling each other and the distribution of smaller size MWCNTs on the surface of SWCNT,a were evident. These results further confirm that the additional amount of MWCNT,b, in this representative case above 10%, or the addition of a small amount of large size CNTs, in this representative case MWCNT,a, contributes to the thickness of buckypaper, instead of filling the pores of resultant buckypaper.

As shown in Figure 7e,f, it was found that the I_D_/I_G_ ratio of buckypapers with and without GFT implementation remained at the same level, instead of increasing because of the higher I_D_/I_G_ ratio of MWCNT,b. These results were in line with the observations of SEM, confirming that 10% MWCNT,b was utilized only to fill the pores in between the layers of SWCNT,a and not distributed on the surface of SWCNT,a. Furthermore, when the amount of MWCNT,b was increased from 10% to 25%, the I_D_/I_G_ ratio was increased because, at the surface of resultant buckypaper, a layer of MWCNT,b was present, in line with the SEM observation, as shown in Figure 7c. Similarly, when it was tried to fill the pores of SWCNT,a-based buckypaper with MWCNT,a (10%), as expected, the resultant buckypaper had a higher I_D_/I_G_ ratio than 100% SWCNT,a-based buckypaper, because, instead of filling the pores, MWCNT,a was added as a separate layer on the surface of resultant buckypaper.

In support of SEM and Raman spectrum observations to validate the improvement in packing density with GFT implementation, AFM was utilized to investigate the surface defects, in terms of surface roughness and peak-to-peak depth. Three randomly selected regions, having an area of 10 µm × 10 µm for each sample were analyzed and summarized in Figure 7g. The experimental data of AFM analysis have been added in supplementary material as Figure S1 and Table S8. Like the observations of SEM and Raman spectrum, the AFM results also confirm that the surface defects in terms of surface roughness and peak-to-peak depth were identical for the buckypapers fabricated with and without GFT implementation. However, the surface defects increased when the 10% MWCNT,b was used as gap filler.

It is necessary to note here, based on the observations made during the validation of the GFT, that the quantity and the dimensions of gap-filler CNTs are critical factors for filling up the pores of buckypaper with the implementation of GFT.

### 3.8. Rule for the Selection of Amount and Size of Gap-Filling CNTs

From a practical perspective, there exists a variety of CNTs differing in their sizes, so there exists a large variety of pores in buckypapers. The application of GFT, especially the determination of quantity and dimension of gap-filler CNTs, can vary for buckypapers. Therefore, it was necessary to develop a “rule of thumb” which can judge the implementation of GFT, so that its application can broaden to all varieties of buckypapers to acquire superior physical properties of resultant buckypaper and its sensors due to enhanced packing density. From this study, it was found that the thickness of buckypaper after the implementation of GFT was the key for the selection of gap-filler CNTs.

The users are not required to perform Raman spectroscopy, SEM, or AFM studies to confirm the GFT implementation. Instead, they are only required to check the thickness of resultant buckypaper after GFT implementation using an optical microscope. Successful selection of gap-filler CNTs and their quantity can easily be judged if the thickness of the consequent buckypaper before and after GFT implementation remains at the same magnitude.

**Figure 8:**
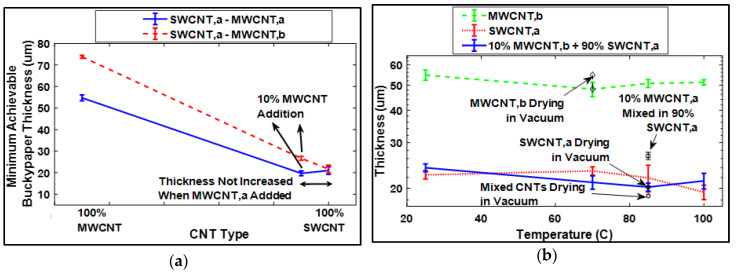
(**a**,**b**) validating that gap-filler CNTs do not contribute to the thickness of resultant buckypaper showing similar thickness for 100% SWCNT,a- and 90% SWCNT,a- and 10% MWCNT,b- (gap filler) based buckypapers.

This fundamental conclusion of GFT was further verified by designing a series of experiments, summarized in tabular form in Table S9 of the supporting materials. In these experiments, the buckypaper thickness was studied by varying the drying conditions after filtration as shown in Figure 8a,b, confirming that, as representative case of this study, 10% MWCNT,b filled the gaps in the SWCNT,a network and did not contribute to the thickness of resultant buckypaper.

### 3.9. Sensor Properties

After filling the pores through the GFT mentioned above, it was necessary to determine the impact of GFT on the physical properties precisely the sensing characteristics of buckypaper. The properties considered in this work were electrical conductivity, GF, tensile strength, and failure strain of buckypaper based sensors. For comparison purposes, these properties were primarily measured for the buckypapers fabricated from 100% SWCNT,a (buckypaper without GFT implementation), 100% MWCNT,b (pure gap-filler buckypaper) and 90% SWCNT,a and 10% MWCNT,b (buckypaper with GFT implementation). To ensure the correctness of the technique, two sets of buckypaper were fabricated for each of the three compositions of buckypaper. These sets were termed as “before post-treatment” and “after post-treatment.” The term “before post-treatment” refers to the buckypaper without washing, and the term “after post-treatment” refers to the buckypaper after washing and heating in a thermal vacuum chamber. The experiments for the investigation of sensor characteristics were performed in lab environment and at room temperature. To ensure the correctness of readings and to avoid human errors, three sample measurements were taken for determining each sensing characteristic. Sensors were made by cutting the buckypaper in a rectangular shape and installed on DMA clamps. Mechanical properties were directly acquired from DMA, and for measuring electrical conductivity and GF, the clamps of DMA, on which buckypaper samples were installed, were connected to digital multimeter having an interface with LabView.

**Figure 9:**
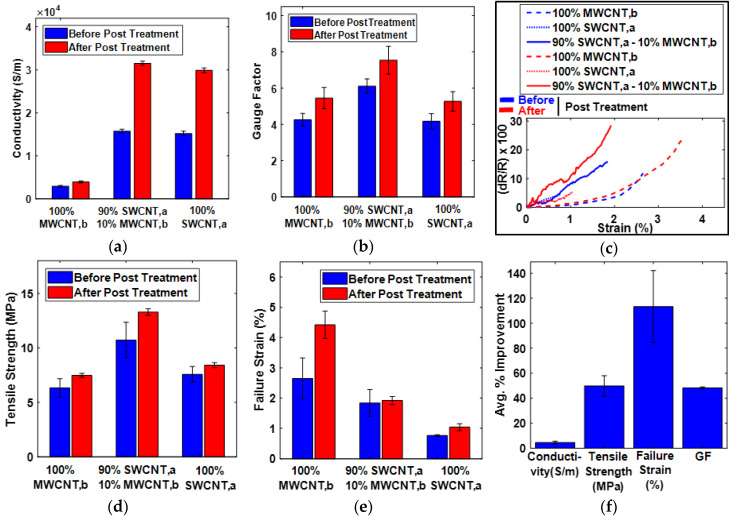
(**a**–**e**) show the physical properties comparison, before and after gap-filling technique (GFT) implementation, showing significant improvement in every considered physical property of the buckypaper after GFT implementation. (**f**) summarizes the percentage improvement in the physical properties of buckypaper after GFT implementation.

The porosity or packing density affects buckypaper properties. Increasing packing density or reducing the pores in buckypaper results in higher inter-tube interactions because of decreased inter-tube distance. The increased inter-tube interactions would directly increase the inter-tube bonding, which increases the stress transfer abilities and interfacial conductivities [33]. In this study of GFT, the pores in SWCNT,a buckypaper, were filled with MWCNT,b as a representative case, so, it was expected to acquire superior properties of sensor developed from buckypaper after GFT implementation. As shown in Figure 9a, the electrical conductivity after GFT implementation was improved. The discontinuity in electrical paths in the form of pores in buckypaper was filled with MWCNT,b (gap-filler), resulting in the improvement of electrical conductivity. As shown in Figure 9b, the increase in the GF of the sensor developed from buckypaper after GFT implementation was evident. The piezoresistivity of buckypaper based sensors was due to the change in contact resistance among individual CNTs upon stretching/loading. The addition of MWCNT,b in the random network of SWCNT,a buckypaper provides additional contacts of CNTs. Upon stretching, these additional contacts provide an incremental change of resistance, resulting in the improvement of GF in the tensile direction. Furthermore, as shown in Figure 9c, the sensitivity of the sensors can be characterized linearly with an increase in the strain magnitude, and the superior sensitivity of the GFT-implemented sensor was evident. However, it can be observe from Figure 9c that the sensor response of a few sensors, especially those which fabricated with 100% MWCNT,b, become non-linear, particularly before reaching failure. For understanding the response non-linearity of CNTs based sensors, we need to imagine the microstructure of the randomly arranged CNTs in buckypaper. The piezoresistivity of buckypaper-based sensors is due to the change in contact resistance among individual CNTs upon stretching/loading. The sensor response becomes severely non-linear just before failing probably because the resistance changes abruptly due to the separation/breakage of inter-tube contacts. Similar results have been obtained in recent studies of carbon-based piezoresistive sensors [59]. The mechanical properties of GFT-implemented sensors, as shown in Figure 9d,e, improved significantly after GFT implementation, due to increased inter-tube interactions resulted from increased packing density of the buckypaper. As shown in Figure 9f, after the implementation of GFT on SWCNT,a buckypaper, the percentage improvement in electrical conductivity, GF, tensile strength and failure strain was calculated to be 4.11 ± 0.61, 44.81 ± 1.72, 49.82 ± 8.21 and 113.36 ± 28.74, respectively. The experimental data of buckypaper-based sensor performance in tabular form have been included in supporting material as Table S10 and the detailed experimental data for conductivity and GF calculations have been included in supporting material as Table S11 and Table S12, respectively.

From Figure 9a, one can observe that the enhancement in electrical conductivity of the buckypaper is only 4% after implementation of GFT, which is far below the improvement in GF (~45%), tensile strength (~50%) and failure strain (~113%). We have tried to investigate the reason behind the small improvement in electrical conductivity and came to the conclusion that after the addition of gap-filler MWCNTs to fill the gaps of SWCNT-based buckypaper, there are two mechanisms introduced in the CNTs microstructure of resultant buckypaper. One is the introduction of additional contacts formed between MWCNTs and SWCNTs, which provide additional paths for the charge transfer, thus increasing the electrical conductivity. Second is the increase in the contact resistance due to additional inter-tube interfaces between gap-filler MWCNTs and SWCNTs, resulting in a decrease in the electrical conductivity. It is likely that, because of these two mechanisms, the net enhancement in electrical conductivity is not comparable to the improvement in GF, tensile strength, and failure strain.

Nevertheless, we can note from the manuscript in Section 2.2 of the “fabrication of buckypaper” that the electrical conductivity of SWCNT-based buckypaper is ~30,000 S/m and of MWCNT-based buckypaper is ~4000 S/m. Based on these conductivity values, one can say that, after mixing, these two CNTs would yield an electrical conductivity of resultant buckypaper less than 30,000 S/m. Instead, after GFT implementation, we are getting electrical conductivity of ~31,200 S/m. The increase in electrical conductivity of resultant buckypaper after GFT implementation, by the addition of MWCNTs having much inferior electrical conductivity, is itself signifying the role of GFT implementation. We believe that more conductive CNTs as gap-filler can enhance the electrical conductivity of resultant buckypaper much higher than 4%.

It can be observed from Figure 9 (and Table S10 of supporting material) that the properties of buckypaper after post-treatment are superior to those before post-treatment. The behavior was such because the moisture contents and amount of surfactant added in the dispersion could have evaporated/decomposed from buckypaper after post-treatment, resulting in improved properties of the buckypaper-based sensors [37,60].

**Figure 10:**
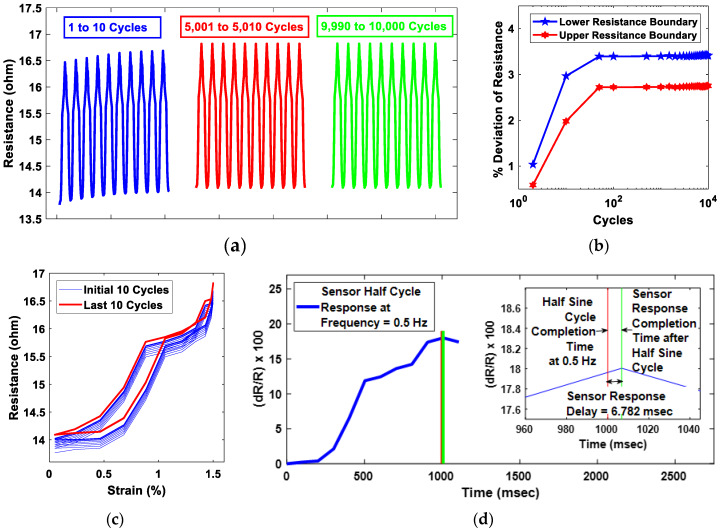
(**a**) shows a 10,000 tensile sine cycles durability test showing stable and repeatable resistance throughout the cycles with exceptions of initial few tens of cycles. (**b**) Cyclic deviation of the upper and the lower boundaries of resistances with respect to initial upper and lower bound resistances. A significant variation can be observed for the first few tens of cycles, after which there was negligible change in resistance boundaries, emphasizing that the GFT-implemented sensor can survive the rigorous dynamic environments. (**c**) Resistance–strain hysteresis comparison between initial and final ten cycles, showing consistent resistance change for the final cycles. (**d**) Sensor response time analysis under a dynamic environment at 0.5 Hz, showing a delay of 6.782 ms.

The durability of the CNT-based sensors can be judged through rigorous cyclic testing [61,62]. In this study, the GFT implemented buckypaper-based sensor was subject to 10,000 cycles sine wave testing in DMA. The amplitude of the sine wave was kept at a constant magnitude of 0.75% strain throughout the 10,000 cycles; thus, the sensor was subjected to an overall strain of 1.5%. The frequency of the sine wave was maintained at 0.5 Hz. As shown in Figure 10a, initially for less than a hundred cycles, the upper and lower resistance of the sensor was climbing cycle-to-cycle, which most probably was the phase of CNTs adjustment in buckypaper under a dynamic environment. To verify the sensor stability under a dynamic environment, the upper and the lower bound resistance change with respect to the very first cycle was analyzed, as shown in Figure 10b. It was found that the upper and the lower boundaries of sensor resistance were climbing initially for a few tens of cycles, resulting in a deviation of ~2.8% in the upper resistance boundary and ~3.5% in the lower resistance boundary. After getting stabilized, the sensor performance was consistent for the rest of the sine wave cycles. Furthermore, the stability of the sensor can be judged by analyzing the resistance–strain hysteresis. As shown in Figure 10c, there was significant variation in hysteresis for the first few cycles, which was because of the deviation of the upper and lower boundary resistances. However, for the final ten cycles, the resistance–strain hysteresis was identical, emphasizing that sensor response was stable at the end of the 10,000 cycle tests. In addition, the sensor response time, which is also considered as a very important parameter for judging the sensor performance, was evaluated through the comparison of the time taken by the sensor to complete a half-sine cycle and the ideal time required to complete the half-sine cycle at 0.5 Hz, i.e., 1000 ms. As shown in Figure 10d, the delay in sensor response to complete the half-sine cycle was estimated to be 6.782 ms, which is comparable to the carbon based piezoresistive sensor response delay mentioned in recent studies [31,32,63].

## 4. Conclusions

The GFT to fill the pores, which can consider as defects, in a randomly arranged CNT network of buckypaper has successfully been established. The gaps in buckypaper fabricated from SWCNTs having a Z-average size of ~377 nm were filled with gap-filler MWCNT having a Z-average size ~63.75 nm. The GFT can easily be employed on any CNT-based buckypapers by carefully selecting the quantity and dimensions of gap-filler CNTs. The selection of gap-filler CNTs can be made easily by examining the thickness of resultant buckypaper in an optical microscope. Such that the selected gap-filler CNTs should not change the thickness of the original buckypaper (with gaps), contribute to increase the packing density of resultant buckypaper only. Through the implementation of GFT, significant improvement in electrical conductivity, tensile GF, tensile strength and failure strain were observed having a percentage improvement of 4.11 ± 0.61, 44.81 ± 1.72, 49.82 ± 8.21 and 113.36 ± 28.74, respectively.

## Supplementary Materials

The following are available online at https://www.mdpi.com/1424-8220/20/10/2904/s1, experimental data and reports of Particle Size Analysis. Figure S1; Table S1–S12.
