# Supplementary material for: A New Route to Enhance the Packing Density of Buckypaper for Superior Piezoresistive Sensor Characteristics
Source: Sensors (Basel). 2020 May 20;20(10):2904. doi: 10.3390/s20102904 (PMC7287720; doi:10.3390/s20102904)
Supplement: Supplementary file 1 [file sensors-20-02904-s001.zip › sensors-780819-supplementary-done/Supporting Material - Experimental Data.docx]

*Supporting Material – Experimental Data*

**A New Route to Enhance the Packing Density of Buckypaper for Superior Piezoresistive Sensor Characteristics**

**Mustafa Danish ^1^ and Sida Luo ^2,^***

^1^ Beihang University, School of Mechanical Engineering & Automation, Beijing, 100191, China; mustafadanish_85@buaa.edu.cn

^2^ Beihang University, School of Mechanical Engineering & Automation, Beijing, 100191, China; s.luo@buaa.edu.cn

***** Correspondence: s.luo@buaa.edu.cn; Tel.: +86-18600200787

**Table S1.** Experimental data of pre-processing dispersion parameters optimization experiments for minimum resistivity of SWCNT buckypaper.

| **Exp. Set** | **Volume of DI Water (ml)** | **SWCNT (mg)** | **Triton X-100 (ml)** | **Sonication Time (hrs)** | **Volume of Dispersion (ml)** | **Centrifuge Time (min)** | **Membrane Pore Size (um)** | **Membrane Material** | **SWCNT Type** | **Resistivity (ohm-mm)** | **Remarks** |
| --- | --- | --- | --- | --- | --- | --- | --- | --- | --- | --- | --- |
| 1 | **100** | 30 | 2.0 | 3 (Pulse 10sec) | 100 | 0 | 5 | PTFE | SWCNT,a | 0.7006 | Initial Parameters |
|  |  | 25 |  |  |  |  |  |  |  | 0.4682 |  |
|  |  | 20 |  |  |  |  |  |  |  | 0.4180 |  |
|  |  | **15** |  |  |  |  |  |  |  | **0.3932** |  |
|  |  | 10 |  |  |  |  |  |  |  | Buckypaper was not successfully peeled off from the membrane | |
| 2 | **100** | **15** | 2.0 | 3 (Pulse 10sec) | 100 | 0 | 5 | PTFE | SWCNT,a | 0.3932 |  |
|  |  |  | **1.5** |  |  |  |  |  |  | **0.3425** |  |
|  |  |  | 1.0 |  |  |  |  |  |  | 0.3756 |  |
| 3 | **100** | **90** | **1.5** | 3 (Pulse 10sec) | 100 | 0 | 5 | PTFE | SWCNT,a | 0.3425 |  |
|  |  |  |  | **6 (Pulse 10sec)** |  |  |  |  |  | **0.3340** |  |
|  |  |  |  | 9 (Pulse 10sec) |  |  |  |  |  | 0.3335 |  |
| 4 | **100** | **90** | **1.5** | **6 (Pulse 10sec)** | 100 | 0 | 5 | PTFE | SWCNT,a | 0.3340 |  |
|  |  |  |  |  | 140 | 10 |  |  |  | 0.3210 |  |
|  |  |  |  |  | **160** | **30** |  |  |  | **0.3047** |  |
|  |  |  |  |  | 180 | 60 |  |  |  | 0.3135 |  |
| 5 | **100** | **90** | **1.5** | **6 (Pulse 10sec)** | 160 | **30** | 5 | PTFE | SWCNT,a | 0.3047 |  |
|  |  |  |  |  | 110 |  | 1 |  |  | 0.1202 |  |
|  |  |  |  |  | 90 |  | 0.45 |  |  | 0.1165 |  |
|  |  |  |  |  | **75** |  | **0.22** |  |  | **0.1041** |  |
| 6 | **100** | **90** | **1.5** | **6 (Pulse 10sec)** | **70** | **30** | **0.22** | PTFE | SWCNT,a | 0.1041 |  |
|  |  |  |  |  |  |  |  | PES |  | 0.1136 |  |
|  |  |  |  |  |  |  |  | **PVDF** |  | **0.0659** |  |
|  |  |  |  |  |  |  | 0.2 | Nylon |  | 0.1209 |  |
|  |  |  |  |  |  |  |  | Al-Oxide |  | 0.0853 |  |
|  |  |  |  |  |  |  |  | MCE |  | 0.0689 |  |
|  |  |  |  |  |  |  |  | PC |  | 0.0949 |  |
| 7 | **100** | **90** | **1.5** | **6 (Pulse 10sec)** | **70** | **30** | **0.22** | **PVDF** | **SWCNT,a** | **0.0659** |  |
|  |  |  |  |  |  |  |  |  | SWCNT,b | 0.7875 |  |

**Table S2.** Experimental data of post-processing drying parameters optimization experiments for minimum resistivity of SWCNT buckypaper.

| **Factor** | **Sample #** | **Factor Level** | **Thickness (um)** | | | **Length (mm)** | **Width (mm)** | **Resistance (ohm)** | **Resistivity (ohm-mm)** | | |
| --- | --- | --- | --- | --- | --- | --- | --- | --- | --- | --- | --- |
|  |  |  | **Sample** | **Avg.** | **Error** |  |  |  | **Sample** | **Avg.** | **Error** |
| **Temperature (C)** | 1 | Room | 21.95 | 22.52 | 0.82 | 15.0 | 2.2 | 21.5 | 0.0692 | 0.0659 | 0.0024 |
|  | 2 |  | 23.68 |  |  | 20.0 | 2.0 | 27.4 | 0.0649 |  |  |
|  | 3 |  | 21.93 |  |  | 25.0 | 3.0 | 24.1 | 0.0636 |  |  |
|  | 1 | 70 | 22.68 | 23.31 | 0.88 | 12.5 | 2.0 | 16.2 | 0.0567 | 0.0549 | 0.0020 |
|  | 2 |  | 24.56 |  |  | 20.5 | 2.2 | 20.6 | 0.0543 |  |  |
|  | 3 |  | 22.68 |  |  | 25.5 | 3.0 | 19.4 | 0.0517 |  |  |
|  | **1** | **85** | **18.42** | **21.93** | **2.58** | **14.0** | **2.4** | **14.3** | **0.0451** | **0.0431** | **0.0014** |
|  | **2** |  | **22.82** |  |  | **21.5** | **2.6** | **15.3** | **0.0422** |  |  |
|  | **3** |  | **24.56** |  |  | **26.2** | **2.7** | **16.6** | **0.0420** |  |  |
|  | 1 | 100 | 18.42 | 19.29 | 1.24 | 12.0 | 2.6 | 10.6 | 0.0423 | 0.0443 | 0.0039 |
|  | 2 |  | 21.05 |  |  | 24.5 | 2.5 | 22.3 | 0.0498 |  |  |
|  | 3 |  | 18.42 |  |  | 28.5 | 3.0 | 21.1 | 0.0409 |  |  |
| **Drying Time (min) (at 85 C)** | 1 | 15 | 21.16 | 20.46 | 0.99 | 15.0 | 1.6 | 22.9 | 0.0517 | 0.0499 | 0.0013 |
|  | 2 |  | 21.16 |  |  | 22.2 | 1.5 | 34.5 | 0.0494 |  |  |
|  | 3 |  | 19.06 |  |  | 27.4 | 2.1 | 33.3 | 0.0487 |  |  |
|  | **1** | **30** | **18.42** | **21.93** | **2.58** | **14.0** | **2.4** | **14.3** | **0.0451** | **0.0431** | **0.0014** |
|  | **2** |  | **22.82** |  |  | **21.5** | **2.6** | **15.3** | **0.0422** |  |  |
|  | **3** |  | **24.56** |  |  | **26.2** | **2.7** | **16.6** | **0.0420** |  |  |
|  | 1 | 45 | 19.04 | 20.20 | 0.82 | 13.0 | 1.8 | 17.7 | 0.0468 | 0.0446 | 0.0024 |
|  | 2 |  | 20.79 |  |  | 21.0 | 2.5 | 18.5 | 0.0457 |  |  |
|  | 3 |  | 20.79 |  |  | 26.0 | 1.6 | 32.3 | 0.0413 |  |  |
|  | 1 | 60 | 18.67 | 17.88 | 0.85 | 16.0 | 2.5 | 15.7 | 0.0458 | 0.0433 | 0.0019 |
|  | 2 |  | 18.28 |  |  | 24.5 | 2.7 | 20.4 | 0.0412 |  |  |
|  | 3 |  | 16.69 |  |  | 27.0 | 3.0 | 23.1 | 0.0428 |  |  |
| **Vacuum Drying (at 85 C for 30 min)** | 1 | No | 18.42 | 21.93 | 2.58 | 14.0 | 2.4 | 14.3 | 0.0451 | 0.0431 | 0.0014 |
|  | 2 |  | 22.82 |  |  | 21.5 | 2.6 | 15.3 | 0.0422 |  |  |
|  | 3 |  | 24.56 |  |  | 26.2 | 2.7 | 16.6 | 0.0420 |  |  |
|  | **1** | **Yes** | **19.30** | **21.64** | **1.80** | **12.5** | **1.9** | **12.4** | **0.0363** | **0.0336** | **0.0020** |
|  | **2** |  | **21.95** |  |  | **20.5** | **2.6** | **11.3** | **0.0314** |  |  |
|  | **3** |  | **23.68** |  |  | **26.0** | **3.0** | **12.1** | **0.0331** |  |  |

**Table S3.** Summary table of SWCNT pre & post-processing parameters optimization experiments for minimum resistivity.

| **Parameter** | **Resistivity (ohm-mm)** | | | |
| --- | --- | --- | --- | --- |
|  | **Sample** | **Value** | **Average** | **Error** |
| Initial | Sample 1 | 0.7234 | 0.7006 | 0.01713 |
|  | Sample 2 | 0.6821 |  |  |
|  | Sample 3 | 0.6963 |  |  |
| SWCNT = 15 mg | Sample 1 | 0.4102 | 0.3932 | 0.01202 |
|  | Sample 2 | 0.3845 |  |  |
|  | Sample 3 | 0.3849 |  |  |
| Triton X-100 = 1.5 ml | Sample 1 | 0.3675 | 0.3425 | 0.01899 |
|  | Sample 2 | 0.3215 |  |  |
|  | Sample 3 | 0.3385 |  |  |
| Sonication Time = 6 hrs, 10 Sec Pulse | Sample 1 | 0.3468 | 0.3340 | 0.01789 |
|  | Sample 2 | 0.3087 |  |  |
|  | Sample 3 | 0.3465 |  |  |
| Centrifuge Time = 30 min, 5000 rpm | Sample 1 | 0.3187 | 0.3047 | 0.01457 |
|  | Sample 2 | 0.2846 |  |  |
|  | Sample 3 | 0.3108 |  |  |
| Membrane Pore Size = 0.22 um | Sample 1 | 0.1189 | 0.1041 | 0.01062 |
|  | Sample 2 | 0.0945 |  |  |
|  | Sample 3 | 0.0989 |  |  |
| Membrane Material = PVDF | Sample 1 | 0.0692 | 0.0659 | 0.00239 |
|  | Sample 2 | 0.0649 |  |  |
|  | Sample 3 | 0.0636 |  |  |
| Drying Temperature = 85 C | Sample 1 | 0.0517 | 0.0499 | 0.00128 |
|  | Sample 2 | 0.0494 |  |  |
|  | Sample 3 | 0.0487 |  |  |
| Drying Time = 30 min | Sample 1 | 0.0451 | 0.0431 | 0.00142 |
|  | Sample 2 | 0.0422 |  |  |
|  | Sample 3 | 0.0420 |  |  |
| Vacuum = Yes | Sample 1 | 0.0363 | 0.0336 | 0.00203 |
|  | Sample 2 | 0.0314 |  |  |
|  | Sample 3 | 0.0331 |  |  |

**Table S4.** Experimental data of pre-processing dispersion parameters optimization experiments for minimum resistivity of MWCNT buckypaper.

| **Exp. Set** | **Volume of DI Water (ml)** | **MWCNT (mg)** | **Triton X-100 (ml)** | **Sonication Time (hrs)** | **Volume of Dispersion (ml)** | **Centrifuge Time (min)** | **Membrane Pore Size (um)** | **Membrane Material** | **MWCNT Type** | **Resistivity (ohm-mm)** | **Remarks** |
| --- | --- | --- | --- | --- | --- | --- | --- | --- | --- | --- | --- |
| 1 | **100** | 200 | 2.0 | 1 (Pulse 5sec) | 100 | 0 | 5 | PTFE | MWCNT,b | 1.5125 | Initial Parameters |
|  |  | 150 |  |  |  |  |  |  |  | 1.4550 |  |
|  |  | 100 |  |  |  |  |  |  |  | 1.3261 |  |
|  |  | **90** |  |  |  |  |  |  |  | **1.2524** |  |
|  |  | 75 |  |  |  |  |  |  |  | Buckypaper was not successfully peeled off from the membrane | |
| 2 | **100** | **90** | 2.0 | 1 (Pulse 5sec) | 100 | 0 | 5 | PTFE | MWCNT,b | 1.2524 |  |
|  |  |  | **1.5** |  |  |  |  |  |  | **0.7387** |  |
|  |  |  | 1.0 |  |  |  |  |  |  | 1.0044 |  |
| 3 | **100** | **90** | **1.5** | **1 (Pulse 5sec)** | 100 | 0 | 5 | PTFE | MWCNT,b | **0.7387** |  |
|  |  |  |  | 2 (Pulse 5sec) |  |  |  |  |  | 0.8233 |  |
|  |  |  |  | 3 (Pulse 5sec) |  |  |  |  |  | 0.9547 |  |
| 4 | **100** | **90** | **1.5** | **1 (Pulse 5sec)** | 100 | 0 | 5 | PTFE | MWCNT,b | 0.7387 |  |
|  |  |  |  |  | 120 | 10 |  |  |  | 0.4765 |  |
|  |  |  |  |  | **130** | **30** |  |  |  | **0.3799** |  |
|  |  |  |  |  | 140 | 60 |  |  |  | 0.3826 |  |
| 5 | **100** | **90** | **1.5** | **1 (Pulse 5sec)** | 130 | **30** | 5 | PTFE | MWCNT,b | 0.3799 |  |
|  |  |  |  |  | 90 |  | 1 |  |  | 0.3691 |  |
|  |  |  |  |  | 80 |  | 0.45 |  |  | 0.3536 |  |
|  |  |  |  |  | **70** |  | **0.22** |  |  | **0.3551** |  |
| 6 | **100** | **90** | **1.5** | **1 (Pulse 5sec)** | **70** | **30** | **0.22** | PTFE | MWCNT,b | 0.3551 |  |
|  |  |  |  |  |  |  |  | PES |  | 0.3516 |  |
|  |  |  |  |  |  |  |  | **PVDF** |  | **0.3424** |  |
|  |  |  |  |  |  |  | **0.2** | Nylon |  | 0.3589 |  |
|  |  |  |  |  |  |  |  | Al-Oxide |  | 0.3695 |  |
|  |  |  |  |  |  |  |  | MCE |  | 0.3486 |  |
|  |  |  |  |  |  |  |  | PC |  | 0.3655 |  |
| 7 | **100** | **90** | **1.5** | **1 (Pulse 5sec)** | **70** | **30** | **0.22** | **PVDF** | MWCNT,b | 0.3424 |  |
|  |  |  |  |  |  |  |  |  | **MWCNT,a** | **0.1234** |  |

**Table S5.** Experimental data of post-processing drying parameters optimization experiments for minimum resistivity of MWCNT buckypaper.

| **Factor** | **Sample #** | **Factor Level** | **Thickness (um)** | | | **Length (mm)** | **Width (mm)** | **Resistance (ohm)** | **Resistivity (ohm-mm)** | | |
| --- | --- | --- | --- | --- | --- | --- | --- | --- | --- | --- | --- |
|  |  |  | **Sample** | **Average** | **Error** |  |  |  | **Sample** | **Average** | **Error** |
| **Temperature (C)** | 1 | Room | 51.32 | 54.74 | 2.49 | 13.0 | 3.4 | 27.9 | 0.3745 | 0.3424 | 0.0233 |
|  | 2 |  | 55.72 |  |  | 22.5 | 3.5 | 38.4 | 0.3328 |  |  |
|  | 3 |  | 57.19 |  |  | 28.5 | 3.6 | 44.3 | 0.3200 |  |  |
|  | **1** | **70** | **51.02** | **48.29** | **3.08** | **13.5** | **2.5** | **32.3** | **0.3052** | **0.2841** | **0.0225** |
|  | **2** |  | **43.99** |  |  | **22.0** | **2.9** | **43.6** | **0.2528** |  |  |
|  | **3** |  | **49.86** |  |  | **25.5** | **2.9** | **51.9** | **0.2943** |  |  |
|  | 1 | 85 | 52.79 | 50.83 | 1.83 | 14.5 | 2.5 | 32.4 | 0.2948 | 0.2832 | 0.00892 |
|  | 2 |  | 48.39 |  |  | 22.5 | 2.5 | 52.4 | 0.2817 |  |  |
|  | 3 |  | 51.32 |  |  | 22.5 | 1.25 | 95.8 | 0.2731 |  |  |
|  | 1 | 100 | 49.86 | 51.32 | 1.20 | 12.5 | 2.1 | 30.3 | 0.2538 | 0.2740 | 0.0144 |
|  | 2 |  | 51.32 |  |  | 17.0 | 2.4 | 38.9 | 0.2818 |  |  |
|  | 3 |  | 52.79 |  |  | 22.5 | 2.8 | 43.6 | 0.2864 |  |  |
| **Drying Time (min) (at 70 C)** | 1 | 15 | 55.72 | 56.21 | 0.69 | 13.5 | 2.3 | 32.1 | 0.3047 | 0.3070 | 0.0025 |
|  | 2 |  | 55.72 |  |  | 19.5 | 2.5 | 42.8 | 0.3057 |  |  |
|  | 3 |  | 57.19 |  |  | 26.0 | 3.2 | 44.1 | 0.3104 |  |  |
|  | **1** | **30** | **51.02** | **48.29** | **3.08** | **13.5** | **2.5** | **32.3** | **0.3052** | **0.2841** | **0.0225** |
|  | **2** |  | **43.99** |  |  | **22.0** | **2.9** | **43.6** | **0.2528** |  |  |
|  | **3** |  | **49.86** |  |  | **25.5** | **2.9** | **51.9** | **0.2943** |  |  |
|  | 1 | 45 | 54.25 | 54.75 | 0.69 | 11.5 | 1.5 | 35.6 | 0.2519 | 0.2833 | 0.0223 |
|  | 2 |  | 54.27 |  |  | 17.5 | 2.8 | 34.3 | 0.2978 |  |  |
|  | 3 |  | 55.72 |  |  | 20.5 | 2.5 | 44.2 | 0.3003 |  |  |
|  | 1 | 60 | 46.92 | 48.39 | 1.20 | 15.5 | 2.4 | 40.8 | 0.2964 | 0.2983 | 0.0019 |
|  | 2 |  | 48.41 |  |  | 17.5 | 2.3 | 47.3 | 0.3009 |  |  |
|  | 3 |  | 49.86 |  |  | 23.0 | 2.6 | 52.8 | 0.2976 |  |  |
| **Vacuum Drying (at 70 C for 30 min)** | 1 | No | 51.02 | 48.29 | 3.08 | 13.5 | 2.5 | 32.3 | 0.3052 | 0.2841 | 0.0225 |
|  | 2 |  | 43.99 |  |  | 22.0 | 2.9 | 43.6 | 0.2528 |  |  |
|  | 3 |  | 49.86 |  |  | 25.5 | 2.9 | 51.9 | 0.2943 |  |  |
|  | **1** | **Yes** | **52.79** | **54.74** | **1.38** | **14.5** | **2.5** | **30.3** | **0.2757** | **0.2552** | **0.0158** |
|  | **2** |  | **55.72** |  |  | **20.5** | **2.2** | **42.3** | **0.2529** |  |  |
|  | **3** |  | **55.72** |  |  | **24.5** | **2.2** | **47.4** | **0.2371** |  |  |

**Table S6.** Summary table of MWCNT pre & post-processing parameters optimization experiments for minimum resistivity.

| **Parameters** | **Resistivity (ohm-mm)** | | | |
| --- | --- | --- | --- | --- |
|  | **Sample** | **Value** | **Average** | **Error** |
| Initial | Sample 1 | 1.6345 | 1.5125 | 0.0896 |
|  | Sample 2 | 1.4218 |  |  |
|  | Sample 3 | 1.4812 |  |  |
| MWCNT = 90 mg | Sample 1 | 1.2987 | 1.2524 | 0.0362 |
|  | Sample 2 | 1.2104 |  |  |
|  | Sample 3 | 1.2481 |  |  |
| Triton X-100 = 1.5 ml | Sample 1 | 0.8765 | 0.7387 | 0.0949 |
|  | Sample 2 | 0.6548 |  |  |
|  | Sample 3 | 0.6848 |  |  |
| Sonication Time = 1 hrs, 5 Sec Pulse | Sample 1 | 0.8765 | 0.7387 | 0.0982 |
|  | Sample 2 | 0.6548 |  |  |
|  | Sample 3 | 0.6848 |  |  |
| Centrifuge Time = 30 min, 5000 rpm | Sample 1 | 0.4512 | 0.3799 | 0.0568 |
|  | Sample 2 | 0.3121 |  |  |
|  | Sample 3 | 0.3764 |  |  |
| Membrane Pore Size = 0.22 um | Sample 1 | 0.4141 | 0.3551 | 0.0473 |
|  | Sample 2 | 0.2984 |  |  |
|  | Sample 3 | 0.3528 |  |  |
| Membrane Material = PVDF | Sample 1 | 0.3745 | 0.3424 | 0.0233 |
|  | Sample 2 | 0.3328 |  |  |
|  | Sample 3 | 0.3200 |  |  |
| Drying Temperature = 70 C | Sample 1 | 0.3047 | 0.3070 | 0.0025 |
|  | Sample 2 | 0.3057 |  |  |
|  | Sample 3 | 0.3104 |  |  |
| Drying Time = 30 min | Sample 1 | 0.3052 | 0.2841 | 0.0225 |
|  | Sample 2 | 0.2528 |  |  |
|  | Sample 3 | 0.2943 |  |  |
| Vacuum = Yes | Sample 1 | 0.2757 | 0.2552 | 0.0158 |
|  | Sample 2 | 0.2529 |  |  |
|  | Sample 3 | 0.2371 |  |  |

**Table S7.** Thickness variation of buckypaper with the addition of gap-filler MWCNT.

| **Buckypaper Composition** | **Buckypaper Thickness (µm)** | | | | |
| --- | --- | --- | --- | --- | --- |
|  | **Sample 1** | **Sample 2** | **Sample 3** | **Average** | **Error** |
| 100 % SWCNT,a | 19.30 | 21.95 | 23.68 | 21.64 | 1.8 |
| 90 % SWCNT,a – 10 % MWCNT.b | 18.56 | 21.28 | 21.84 | 20.56 | 1.43 |
| 90 % SWCNT,a – 10 % MWCNT.a | 26.71 | 28.34 | 28.80 | 27.95 | 0.89 |
| 75 % SWCNT,a – 25 % MWCNT.b | 28.58 | 30.16 | 32.22 | 30.32 | 1.49 |
| 50 % SWCNT,a – 50 % MWCNT.b | 33.29 | 37.17 | 38.92 | 36.46 | 2.35 |
| 25 % SWCNT,a – 75 % MWCNT.b | 40.67 | 42.48 | 47.08 | 43.41 | 2.69 |
| 100 % MWCNT.b | 52.79 | 55.72 | 55.72 | 54.74 | 1.38 |

| 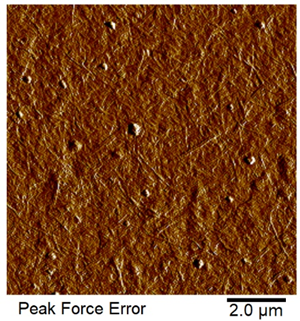  (**a**) | 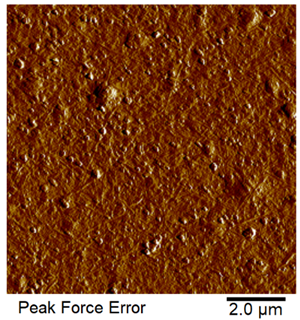  (**b**) | 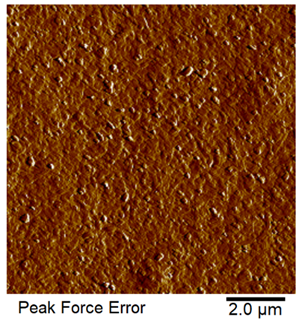(**c**) |
| --- | --- | --- |
| 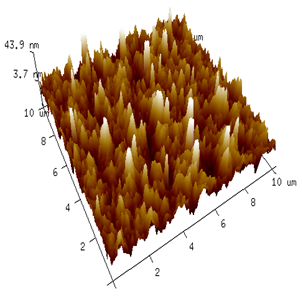  (**d**) | 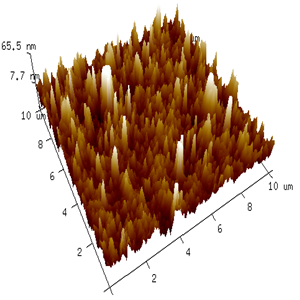  (**e**) | 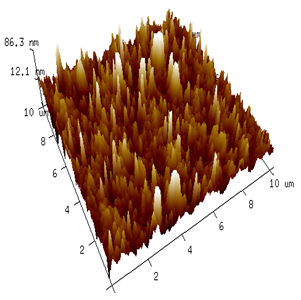(**f**) |
| 100 % SWCNT,a Buckypaper | 90 % SWCNT,a – 10% MWCNT,b Buckypaper | 90 % SWCNT,a – 10% MWCNT,a Buckypaper |

**Figure S1.** (a), (b) & (c) AFM surface morphology of buckypaper for mentioned CNTs compositions. Surface morphology with & without GFT implementation resembling each other. (d), (e) & (f) AFM topography of buckypaper for mentioned CNT compositions, showing maximum height.

**Table S8.** Experimental data of buckypaper surface roughness obtained from AFM analysis.

| **Buckypaper Composition** | **Sample** | **Roughness Rq (nm)** | | | **Roughness Ra (nm)** | | | **Peak – Peak Depth (nm)** | | |
| --- | --- | --- | --- | --- | --- | --- | --- | --- | --- | --- |
|  |  | **Value** | **Average** | **Error** | **Value** | **Average** | **Error** | **Value** | **Average** | **Error** |
| 100 % SWCNT,a | S1 | 15.6 | 15.7 | 0.82 | 12.1 | 11.9 | 0.34 | 1.33 | 2.09 | 0.75 |
|  | S2 | 16.8 |  |  | 12.3 |  |  | 1.82 |  |  |
|  | S3 | 14.8 |  |  | 11.5 |  |  | 3.12 |  |  |
| 90 % SWCNT,a – 10% MWCNT,b | S1 | 17.4 | 19.4 | 2.17 | 12.2 | 13.1 | 0.87 | 3.17 | 2.30 | 0.62 |
|  | S2 | 18.3 |  |  | 12.9 |  |  | 1.73 |  |  |
|  | S3 | 22.4 |  |  | 14.3 |  |  | 2.01 |  |  |
| 90 % SWCNT,a – 10% MWCNT,a | S1 | 51 | 41.7 | 7.76 | 41 | 32.7 | 6.01 | 5.81 | 4.50 | 1.62 |
|  | S2 | 42 |  |  | 30 |  |  | 5.48 |  |  |
|  | S3 | 32 |  |  | 27 |  |  | 2.21 |  |  |

**Table S9.** Buckypaper thickness variation with temperature.

| **Temperature (C)** | **Buckypaper Thickness (µm)** | | | | | | | | | | | | | | | |
| --- | --- | --- | --- | --- | --- | --- | --- | --- | --- | --- | --- | --- | --- | --- | --- | --- |
|  | **MWCNT,b** | | | | **SWCNT,a** | | | | **90 % SWCNT,a - 10 % MWCNT,b** | | | | **90 % SWCNT,a - 10 % MWCNT,a** | | | |
|  | **Without Vacuum** | | **With Vacuum** | | **Without Vacuum** | | **With Vacuum** | | **Without Vacuum** | | **With Vacuum** | | **Without Vacuum** | | **With Vacuum** | |
|  | **Value** | **Error** | **Value** | **Error** | **Value** | **Error** | **Value** | **Error** | **Value** | **Error** | **Value** | **Error** | **Value** | **Error** | **Value** | **Error** |
| Room | 54.74 | 2.49 |  |  | 22.52 | 0.82 |  |  | 23.99 | 0.83 |  |  |  |  |  |  |
| 70 | 48.29 | 3.08 | 54.74 | 1.38 | 23.31 | 0.88 |  |  | 21.06 | 1.25 |  |  |  |  |  |  |
| 85 | 50.83 | 1.83 |  |  | 21.93 | 2.58 | 21.64 | 1.80 | 20.19 | 0.72 | 20.56 | 1.43 |  |  | 27.95 | 0.89 |
| 100 | 51.32 | 1.20 |  |  | 19.29 | 1.24 |  |  | 21.34 | 1.49 |  |  |  |  |  |  |

**Table S10.** Buckypaper sensor performance evaluation with and without GFT implementation

|  |  | **Before GFT Implementation** | | **After GFT Implementation** | |
| --- | --- | --- | --- | --- | --- |
|  |  | **Before Washing** | **After Washing** | **Before Washing** | **After Washing** |
| **CNTs** | **Volume (%)** | SWCNT = 100% | | SWCNT = 90%  MWCNT = 10% | |
|  | **Mass (mg)** | SWCNT = 11.25 | | SWCNT = 10.12  MWCNT = 6.3 | |
| **Buckypaper** | **Thickness (µm)** | 22.41 ± 1.6 | 21.64 ± 1.8 | 23.96 ± 0.8 | 20.56 ± 1.1 |
|  | **Mass (mg)** | 23.1 ± 2.6 | 14.5 ± 0.3 | 35.5 ± 2.3 | 20.1 ± 0.8 |
|  | **Density (kg/m3)** | 820.3 ± 138 | 533.2 ± 69 | 1179.1 ± 104 | 778.1 ± 44 |
| **Sensor Performance Parameters** | **Gauge Factor** | 4.07 ± 0.40 | 5.27 ± 0.53 | 6.11 ± 0.57 | 7.61 ± 0.89 |
|  | **Failure Strain (%)** | 0.76 ± 0.03 | 1.04 ± 0.11 | 1.84 ± 0.44 | 1.92 ± 0.13 |
|  | **Tensile Strength (MPa)** | 7.57 ± 0.71 | 8.41 ± 0.23 | 10.72 ± 1.63 | 13.26 ± 0.29 |
|  | **Electrical Conductivity (S/m)** | 15,200 ± 540 | 29,800 ± 570 | 15,720 ± 450 | 31,200 ± 480 |

**Table S11.** Experimental data of conductivity calculations

| **Without GFT Implementation (100% SWCNT,a Based Buckypaper)** | | | | | | | | | | | |
| --- | --- | --- | --- | --- | --- | --- | --- | --- | --- | --- | --- |
| **Before Post Treatment** | | | | | | | | | | | |
| **Sample #** | **Thickness (um)** | | **Length (mm)** | | **Width (mm)** | | **Resistance (ohm)** | **Resistivity (ohm-mm)** | **Conductivity (S/m)** | **Avg. Conductivity (S/m)** | **Error (S/m)** |
| 1 | 21.95 | | 15 | | 2.2 | | 21.5 | 0.0692 | 14450.86 | 15194.15 | 541.08 |
| 2 | 23.68 | | 20 | | 2 | | 27.4 | 0.0649 | 15408.32 |  |  |
| 3 | 21.93 | | 25 | | 3 | | 24.1 | 0.0636 | 15723.27 |  |  |
| **After Post Treatment** | | | | | | | | | | | |
| 1 | 23.68 | | 26 | | 3 | | 12.1 | 0.0331 | 30211.48 | 29426.14 | 566.15 |
| 2 | 22.18 | | 24.5 | | 3.2 | | 11.9 | 0.0346 | 28905.51 |  |  |
| 3 | 22.97 | | 28.5 | | 3.6 | | 11.8 | 0.0343 | 29154.52 |  |  |
| **With GFT Implementation (90% SWCNT,a – 10% MWCNT,b Based Buckypaper)** | | | | | | | | | | | |
| **Before Post Treatment** | | | | | | | | | | | |
| 1 | 24.58 | 13.5 | | 2.5 | | 14.1 | | 0.0642 | 15576.32 | 15727.97 | 450.65 |
| 2 | 24.58 | 21.5 | | 2.6 | | 20.6 | | 0.0612 | 16339.87 |  |  |
| 3 | 22.81 | 26.5 | | 2.8 | | 27.2 | | 0.0655 | 15267.71 |  |  |
| **After Post Treatment** | | | | | | | | | | | |
| 1 | 18.91 | 24.8 | | 2.6 | | 15.8 | | 0.0313 | 31924.91 | 31274.33 | 482.84 |
| 2 | 19.62 | 23.4 | | 2.9 | | 13.2 | | 0.0321 | 31154.56 |  |  |
| 3 | 19.47 | 28.6 | | 2.5 | | 19.1 | | 0.0325 | 30762.54 |  |  |

**Table S12.** Experimental data for GF calculations

| **Without GFT Implementation (100% SWCNT,a Based Buckypaper)** | | | | |
| --- | --- | --- | --- | --- |
| **Before Post Treatment** | | | | |
| **Sample #** | **Avg. Failure Strain (%)** | **GF at Avg. Strain %** | **Avg. GF** | **Error** |
| 1 | 0.76 | 3.71 | 4.06 | 0.39 |
| 2 |  | 4.62 |  |  |
| 3 |  | 3.864 |  |  |
| **After Post Treatment** | | | | |
| 1 | 1.04 | 5.98 | 5.27 | 0.53 |
| 2 |  | 4.69 |  |  |
| 3 |  | 5.14 |  |  |
| **With GFT Implementation (90% SWCNT,a – 10% MWCNT,b Based Buckypaper)** | | | | |
| **Before Post Treatment** | | | | |
| 1 | 1.84 | 5.15 | 6.11 | 0.57 |
| 2 |  | 6.65 |  |  |
| 3 |  | 6.25 |  |  |
| 4 |  | 6.41 |  |  |
| **After Post Treatment** | | | | |
| 1 | 1.92 | 6.56 | 7.61 | 0.89 |
| 2 |  | 7.88 |  |  |
| 3 |  | 8.94 |  |  |
| 4 |  | 7.08 |  |  |
