# Supplementary material for: A New Route to Enhance the Packing Density of Buckypaper for Superior Piezoresistive Sensor Characteristics
Source: Sensors (Basel). 2020 May 20;20(10):2904. doi: 10.3390/s20102904 (PMC7287720; doi:10.3390/s20102904)

Size Distribution Report by Intensity

v2.2

Sample Details

**Sample Name:**	1 1
**SOP Name:**	mansettings.nano
**General Notes:**	

**File Name:**	11.dts	**Dispersant Name:**	Water
**Record Number:**	18	**Dispersant RI:**	1.330
**Material RI:**	1.59	**Viscosity (cP):**	0.8872
**Material Absorbtion:**	0.010	**Measurement Date and Time:**	2019年11月6日 13:59:09

System

**Temperature (°C):**	25.0	**Duration Used (s):**	50
**Count Rate (kcps):**	137.6	**Measurement Position (mm):**	4.65
**Cell Description:**	Disposable sizing cuvette	**Attenuator:**	11

Results

			**Size (r.nm...**	**% Intensity:**	**St Dev (r.n...**
**Z-Average (r.nm):**	377.2	**Peak 1:**	416.7	81.5	96.09
**PdI:**	0.787	**Peak 2:**	70.14	18.5	11.94
**Intercept:**	0.929	**Peak 3:**	0.000	0.0	0.000

Result quality **Refer to quality report**

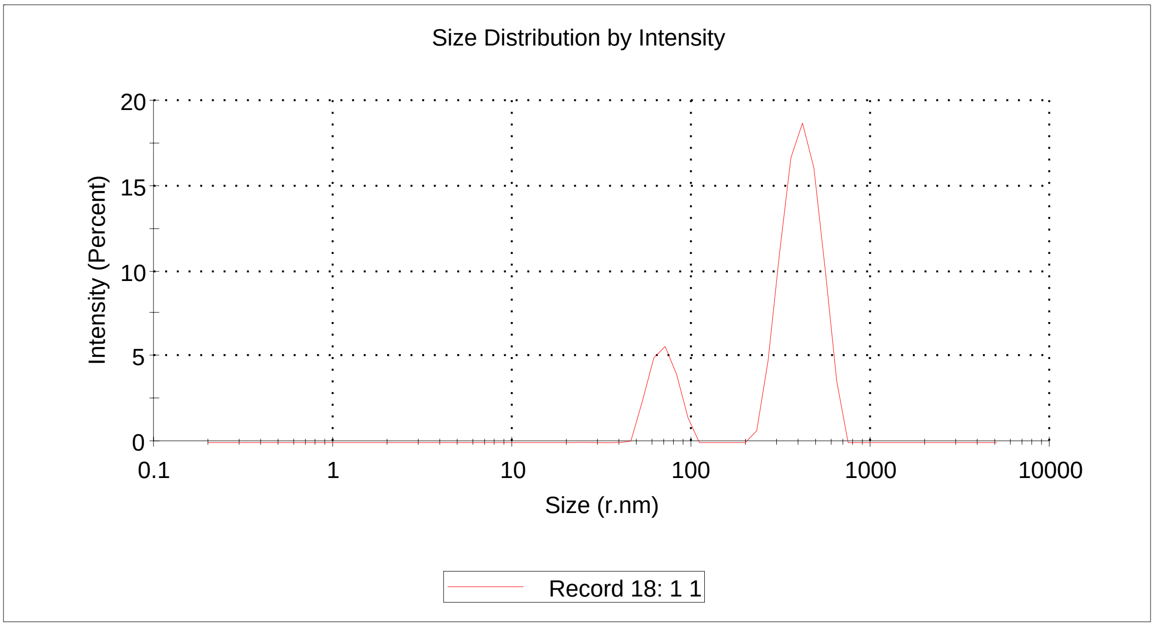

Supplement: Supplementary file 1 [file sensors-20-02904-s001.zip › sensors-780819-supplementary-done/Supporting Material - PSA Reports.zip!/SWCNT,a.pdf]
